# Analysis of Effects of Surface Roughness on Sensing Performance of Surface Plasmon Resonance Detection for Refractive Index Sensing Application

**DOI:** 10.3390/s21186164

**Published:** 2021-09-14

**Authors:** Treesukon Treebupachatsakul, Siratchakrit Shinnakerdchoke, Suejit Pechprasarn

**Affiliations:** 1 Department of Biomedical Engineering, School of Engineering, King Mongkut’s Institute of Technology Ladkrabang, Bangkok 10520, Thailand; treesukon.tr@kmitl.ac.th (T.T.); 61011333@kmitl.ac.th (S.S.); 2 College of Biomedical Engineering, Rangsit University, Pathum Thani 12000, Thailand

**Keywords:** surface plasmon resonance, sensing performance, refractive index sensing, surface roughness, instrumentation

## Abstract

This paper provides a theoretical framework to analyze and quantify roughness effects on sensing performance parameters of surface plasmon resonance measurements. Rigorous coupled-wave analysis and the Monte Carlo method were applied to compute plasmonic reflectance spectra for different surface roughness profiles. The rough surfaces were generated using the low pass frequency filtering method. Different coating and surface treatments and their reported root-mean-square roughness in the literature were extracted and investigated in this study to calculate the refractive index sensing performance parameters, including sensitivity, full width at half maximum, plasmonic dip intensity, plasmonic dip position, and figure of merit. Here, we propose a figure-of-merit equation considering optical intensity contrast and signal-to-noise ratio. The proposed figure-of-merit equation could predict a similar refractive index sensing performance compared to experimental results reported in the literature. The surface roughness height strongly affected all the performance parameters, resulting in a degraded figure of merit for surface plasmon resonance measurement.

## 1. Introduction

Surface plasmons are oscillations due to moving electrons that exist at an interface between conductors and dielectrics [1]. This phenomenon can generate a resonating effect when illuminated by an external electric field called surface plasmon resonance (SPR) [2]. The theory of SPR has been utilized for numerous sensing technology and applications, including host–pathogen detection [3,4], protein interactions [5,6], ultrasonic detections [7], refractive index sensing [8,9], voltage sensing [10], and microscopic imaging application [11,12]. For example, Lan et al. [13] reported the binding kinetics of SARS-CoV-2 and angiotensin-converting enzyme 2 (ACE2) using SPR measurement.

**Figure 1:**
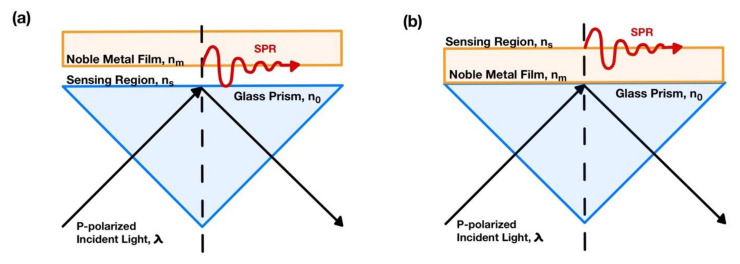
(**a**) The Otto configuration; and (**b**) the Kretschmann configuration.

The surface plasmon polariton (SPP)-based sensor can be classified into two primary configurations: Otto [14] and Kretschmann [15] configurations, as shown in Figure 1a,b [6]. A beam of p-polarized coherent incident light penetrates through a glass prism, illuminates the thin noble metal surface, and reflects off the metal surface.

**Figure 2:**
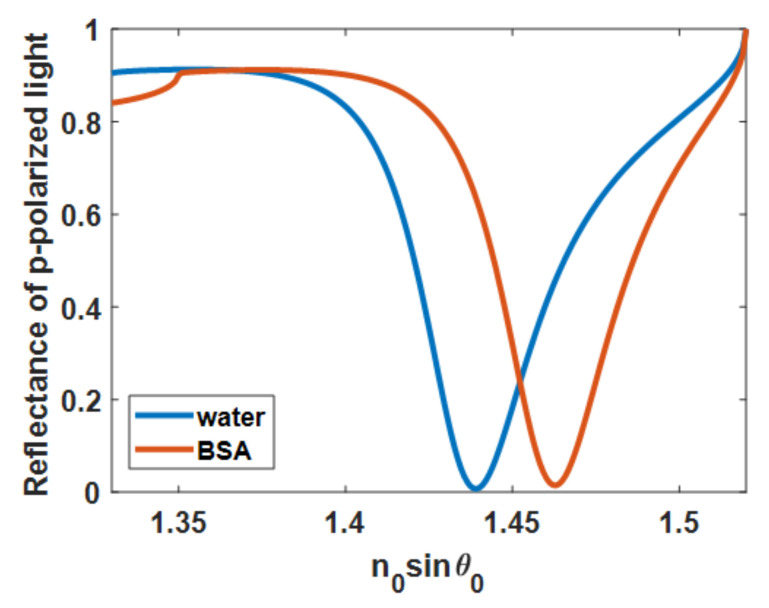
Reflectance spectra of uniform 50 nm gold on BK7 glass substrate when the gold sensor was illuminated by p-polarized coherent light at 633 nm. The blue curve showed the reflectance spectrum when the sample sensing region was water, and the red curve showed the reflectance spectrum when the sample sensing region was BSA protein solution with a sample refractive index of 1.35.

Figure 2 depicts reflectance dips, called surface plasmon resonance dips, and the minimum position of the dip is called plasmonic angle *θ_sp_* due to the coupling process [16] and the quantum light–matter interaction between photons and electrons [17]. The *θ_sp_* position is sensitive to changes in external conditions around the metal surface, such as the refractive index of surrounding media, noble metal thickness, and incident light wavelength. These enable numerous label-free sensing technologies [18,19]. The blue and red curves in Figure 2 were calculated using rigorous coupled-wave theory [20,21] for a water-based environment with a refractive index of the sensing region *n_s_* of 1.33, bovine serum albumin (BSA) at a concentration of 80 mg/mL with a refractive index *n_s_* of 1.35 [22].

The difference between the Kretschmann configuration and the Otto configuration is that the Otto configuration requires a narrow spacing gap; the gap is typically a wavelength or subwavelength thickness. Although Shen et al. [8] and Pechprasarn et al. [23] reported that the Otto configuration could provide higher sensitivity, the gap has burdened fabrication demand. As a result, SPR sensors usually employ the Kretschmann configuration and have become more commonly known as the traditional SPR-based sensor [24]. The effect of surface roughness for the Kretschmann configuration and Otto configuration is similar; hence, the Otto result was omitted to shorten the length of the manuscript.

Smooth plasmonic sensors can be achieved through chemical polishing [25,26], using mica substrate [27], the stripping method [28,29], self-limiting galvanic displacement [30], chemically grown single-crystalline gold [31], laser ablation [32], helium ion beam [33], and thermal annealing [34]. Table 1 shows the remaining roughness of the plasmonic gold sensor after different surface treatments.

For sensitivity-demanding applications, such as single-molecule detection [35] and small-molecule measurements [36], it is established that the smoothness and uniformity of plasmonic metal play a crucial role in sensing performance [37,38,39,40] and electrical conductivity [41]. In addition, the roughness can introduce additional ohmic loss to the surface wave attenuation coefficient [11].

Theoretical papers on SPR sensors [35,42,43] have reported the theoretical sensitivity for SPR calculated using ideally smooth metal surfaces. In addition, the effects of roughness on SPR characteristics, resonant conditions [44] and reflectance [45], transmittance, and absorption spectra [46] have been reported and investigated. For example, research groups have proposed a dispersion-relation model [45,47] and measured a rough-surface plasmon sensor’s propagation length [48]. In addition, Yang et al. [49] experimentally validated that the plasmonic angle depended on the roughness of the metal film using the surface-annealing method.

For sensing applications, the effect of roughness on the sensitivity is established [50,51]: the roughness can degrade the bulk sensitivity; however, it also can enhance the binding sensitivity due to the enhanced localized surface plasmon and an increased surface area for the chemical reaction [52]. Byun et al. [52] proposed a theoretical model quantifying the sensitivity for different roughness using a binary grating model. However, the sensitivity alone cannot provide a complete justification of the sensing performance since the sensitivity does not consider the quality factor (Q factor) describing how narrow the plasmonic dip is [53].

The current trend and the state-of-the-art technology for SPR measurement are to measure smaller biological molecule size [54,55] and numbers of molecules [56,57], aiming towards single-molecule detection [58]. In addition, SPR measurement methods have been proposed to enhance the sensitivity in SPR measurement, including phase detection [59,60], long-range surface plasmon polaritons [61], and metamaterial surfaces [62,63]. There are, of course, challenges in achieving such measurements, including environmental stability and the quality of the plasmonic sensor surface. This study has excluded all the other factors and quantified the effects of the sensor’s surface roughness, providing an indicator for estimating the expected SPR sensing performance.

This research aims to provide a theoretical framework to analyze and quantify the effects of surface roughness of the surface plasmon resonance sensor on sensing performance parameters, including sensitivity, optical intensity at the plasmonic angle, position of plasmonic dips, full width at half maximum (FWHM), and figure of merit (FOM) using rigorous coupled-wave analysis and Monte Carlo simulations. Thus, the relevant sensing parameters provide an insight into the effects of roughness on the bulk refractive index measurement performance. In addition, we believe the paper provides a complete assessment of how the roughness affects the bulk refractive index sensing measurement and how far from the theoretical limit can be expected for a plasmonic sensor with a roughness range.

## 2. Materials and Methods

### 2.1. Surface Plasmon Resonance Detection and Rough Surface Model

**Figure 3:**
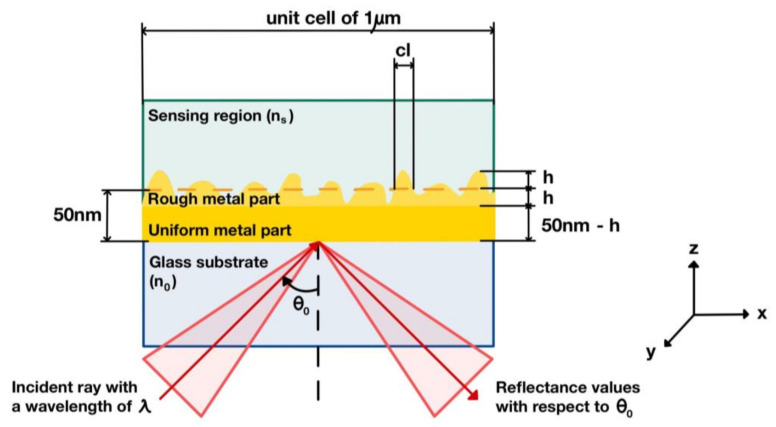
Simulation diagram including roughness parameters required for constructing rough surface profiles.

The simulation structure, as illustrated in Figure 3, consisted of (1) a p-polarized helium–neon laser (HeNe) source at 633 nm wavelength *λ*, (2) a glass substrate made of BK7 glass with a refractive index of 1.52, and (3) uniform and rough gold layers with a refractive index of 0.18344 + 3.4332i [64] and average thicknesses of 50 nm–*h* and *h*, respectively. The h term is the arithmetic mean thickness of the rough surfaces. A unit cell length of 1 μm was chosen and represented 1000 pixels based on the sampling theory to ensure that the unit cell accommodated different roughnesses, ranging from the minimum of 1 nm to the maximum of 50 nm. For the 50 nm case, the unit cell of 1 μm had 20 different roughness peaks, mimicking a genuine surface-roughness nature.

**Figure 4:**
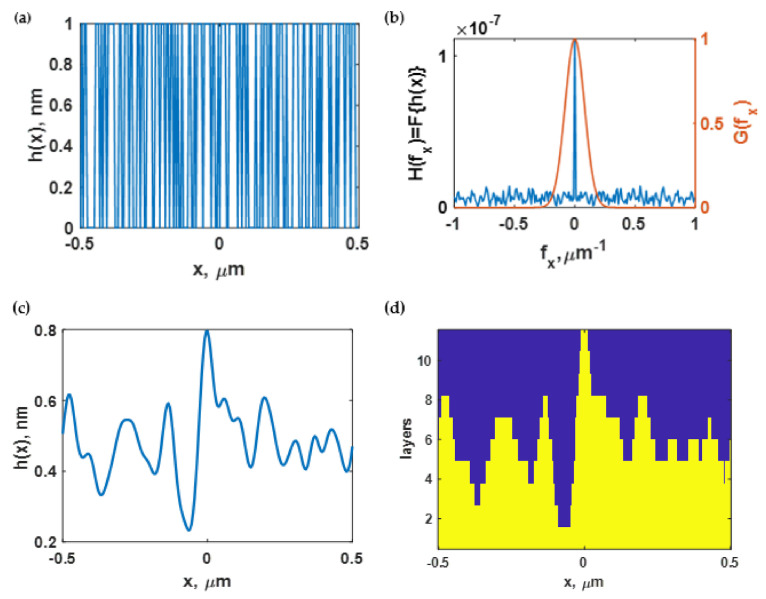
(**a**) Product of *h* and a sequence of randomized 0 and 1; (**b**) magnitude of *H(f_x_)* after Fourier transform (indicated as the blue curve) and a Gaussian distribution plot determined by *cl* (shown as the red curve); (**c**) inverse Fourier transform of the product $H\left(f_{x}\right)\cdot G\left(f_{x}\right)$, resulting in a rough surface structure; and (**d**) the 10-layer rough gold surface and a single-layer uniform gold surface.

The overall metal layer thickness was also fixed at 50 nm. The 50 nm gold layer was an optimum gold thickness for a sensing application since it gives the lowest intensity plasmonic dip [65]. The roughness was defined using two parameters: the average height of the rough surface *h* and the correlation length *cl*. This study’s range of *h* and *cl* was 1 nm to 20 nm and 1 nm to 50 nm, respectively. The range of *h* and *cl* were chosen so that the roughness profiles covered the conventional SPR excitation and the SPR mode cut-off positions for the two variables, as shown later in the Results section. The following steps adopted from Byun et al. [52] are for simulating the rough surface profiles:

(1) Generate a random surface profile $h\left(x\right)$ with digital numbers 0 and 1 and multiply by the expected h, where x is the spatial distance along the *x*-axis of the substrate, as depicted in Figure 4a.
(2) Fourier transform the generated surface $H\left(f_{x}\right)=F\left[h\left(x\right)\right]$, where $H\left(f_{x}\right)$ is the Fourier-transformed profile, *f_x_* is the Fourier domain axis, and F is the Fourier-transform operator, as depicted in Figure 4b.
(3) Low-pass filter the Fourier surface profile using the Gaussian distribution function $G\left(f_{x}\right)$ expressed as shown in Equation (1) and depicted by red curves in Figure 4b.
  (1) $$G\left(f_{x}\right)=exp\left(-x^{2}/\left(cl^{2}/2\right)\right)$$
(4) Inverse Fourier transform the product of $F^{-1}\left\{H\left(f_{x}\right)\cdot G\left(f_{x}\right)\right\}$ to obtain the rough surface profile, as depicted in Figure 4c.

The other approach to define the roughness is the root mean square (*RMS*) of the surface profile given by Equation (2):

(2) $$RMS=\sqrt{\overset{0.5\mu m}{\underset{-0.5\mu m}{\int }}{\left[h\left(x\right)-h\right]}^{2}dx}$$

The rough surface profile was then converted into the rough gold surface, represented by 10 layers for rigorous coupled-wave analysis calculation. The surface profile was modeled in a rectangular coordinate system. In addition, the area below the surface profile was the gold material with the refractive index of 0.18344 + 3.4332i [64], while the upper area was the sensing region with a refractive index of *n_s_* shown in Figure 4d. Finally, the constructed rough surface was assembled with a uniform gold layer thickness of 50 nm–*h* before proceeding with the optical-simulation process.

### 2.2. Optical Simulation Using Rigorous Coupled-Wave Analysis

Rigorous coupled-wave analysis (RCWA) [21,66] was employed to compute the reflectance spectrum of the SPR detection platforms for different rough surfaces. The RCWA software was developed in-house in the MATLAB environment utilizing the parallel computing and graphic processing toolboxes. All the simulations reported in the manuscript were simulated using 151 diffraction orders to ensure that all the cases achieved convergence. The numerical stability and accuracy for the 151 diffracted orders were within 0.0028, corresponding to a numerical error of 0.28%. The convergent tests for all the extreme cases in the manuscript are reported and discussed later in Section 3.1.

**Figure 5:**
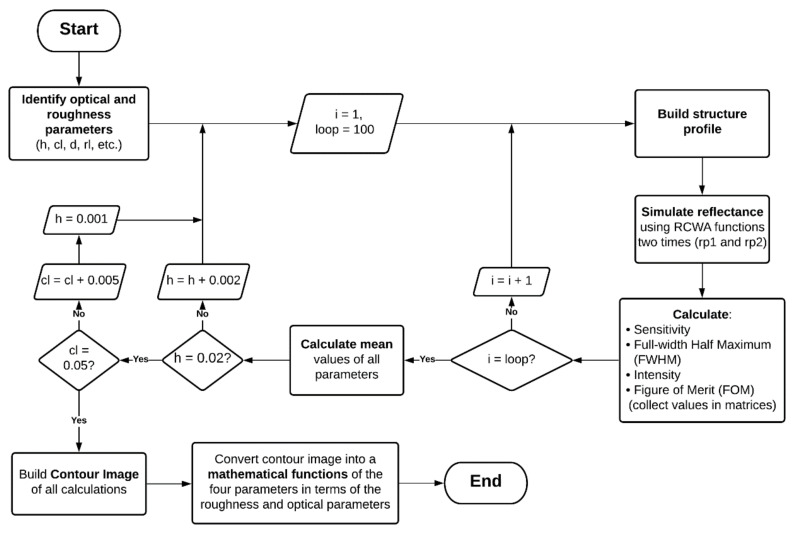
Flowchart of the simulation and calculation processes, including structure profile construction, RCWA, and sensor-quality computation.

Since the rough surfaces were generated randomly, Monte Carlo simulation, which is very effective for estimating the results from an uncertain event [67], was applied in this study. The computation proceeded with the RCWA simulation 100 times per one set of roughness parameters as depicted by the process flow in Figure 5 to ensure that the recovered quantitative parameters described in the next section were stable for any single pair of height *h* and correlation length *cl*.

### 2.3. Quantitative Performance Parameters

The RCWA simulation was applied to compute optical reflectance responses for the sensing region *n_s_* with a refractive index of 1.33 (water) and 1.35 (bovine serum albumin protein solution) [68] to quantify the bulk sensitivity response of the rough surfaces. The quantitative performance parameters were: sensitivity (*S*), full width at half maximum (*FWHM*), intensity difference (Δ*I*), dip intensity at the plasmonic angle (*I_sp_*), and figure of merit (*FOM*).

**Figure 6:**
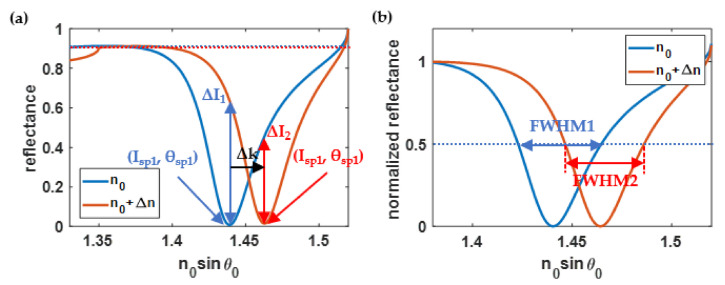
(**a**) Calculation methods of all quantitative performance parameters of the surface plasmon resonance detection; and (**b**) normalized optical reflectance and the full width at half maximum (FWHM).

(1) Sensitivity (*S*) was defined as the change in plasmonic wave vector (*k_sp_*) over the change in refractive index (*n_s_*) in the sensing region, as depicted in Figure 6a and expressed in Equation (3). The unit of *S* is rad· RIU^−1^/μm. Note that RIU stands for refractive index unit.

(3) $$S=\frac{\Delta k_{sp}}{\Delta n_{s}}=\frac{2\pi n_{0}\Delta sin\theta_{sp}}{\lambda \Delta n_{s}}$$

(2) The full width at half maximum (*FWHM*) was defined as the average width of the SPR dips with the *n_s_* of 1.33 and 1.35 cases in wave-vector space with an optical intensity of at least 0.5 (50%) of the normalized optical reflectance spectra, as depicted in Figure 6b and expressed in Equation (4). Thus, the unit of the *FWHM* is rad/μm.
  (4) $$FWHM=\frac{\Delta k_{nor,1.33}+\Delta k_{nor,1.35}}{2}$$
  where $\Delta k_{nor,1.33}$ and $\Delta k_{nor,1.35}$ are the *FWHM* of the normalized SPR reflectance spectra when the refractive index of the sensing region was 1.33 and 1.35, respectively.
(3) Intensity contrast (ΔI) was the average change in optical reflectance at the two plasmonic dips, as shown in Figure 6a and expressed by Equation (5):
  (5) $$\Delta I=\frac{\left|\Delta I_{1.33}\left|+\right|\Delta I_{1.35}\right|}{2}$$
  where $\Delta I_{1.33}$ and $\Delta I_{1.35}$ are the change in optical reflectance at the plasmonic angles when the refractive index of the sensing region was 1.33 and 1.35, respectively.
(4) Optical reflectance at the plasmonic angle ($I_{sp}$) was defined as the average optical reflectance ($I_{sp1.33}$ and $I_{sp1.35}$) of the two plasmonic dips when the refractive index of the sensing region was 1.33 and 1.35, as expressed in Equation (6):

(6) $$I_{sp}=\frac{I_{sp1.33}+I_{sp1.35}}{2}$$

(5) The figure of merit (*FOM*) was defined by considering the dip movement, the *FWHM*, and the intensity level. Here, the *FOM* was defined as shown in Equation (7):

(7) $$FOM=\frac{\Delta I\times S}{I_{sp}\times \mathrm{FWHM}}=\frac{\Delta I\times \Delta k_{sp}}{I_{sp}\Delta n_{s}\mathrm{FWHM}}$$

## 3. Results and Discussion

### 3.1. Convergence Test of Extreme Cases

**Figure 7:**
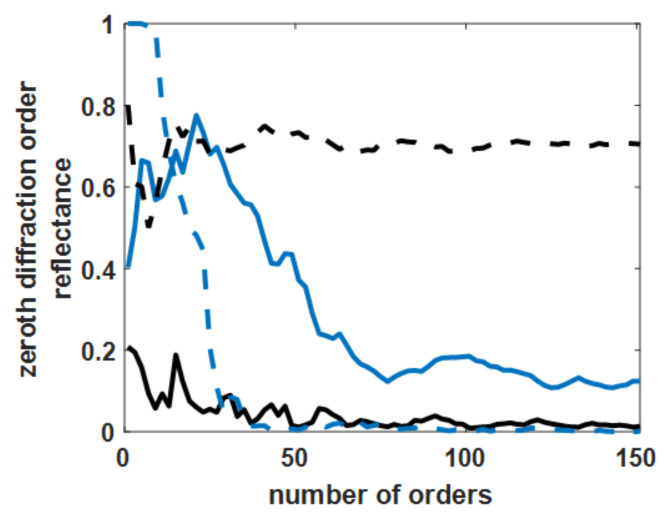
Optical reflectance of the p-polarization for four levels of rough surfaces with the varying number of diffracted orders included in the RCWA calculations. Note that the solid blue curve is for *cl* of 1 nm and *h* of 1 nm, the dashed blue curve is for *cl* of 1 nm and *h* of 20 nm, the solid black curve is for *cl* of 50 nm and *h* of 1 nm, and the dashed blue curve is for *cl* of 50 nm and *h* of 20 nm.

The roughness range studied was the *cl* of 1 nm to 50 nm and the *h* of 1 nm to 20 nm. Therefore, the four boundary cases were: (1) *cl* of 1 nm and *h* of 1 nm; (2) *cl* of 1 nm and *h* of 20 nm; (3) *cl* of 50 nm and *h* of 1 nm; and (4) *cl* of 50 nm and *h* of 20 nm. Figure 7 shows the convergence test by varying the number of diffracted orders included in the RCWA calculations using the p-polarized wave at a 633 nm wavelength, and the incident angle was at the plasmonic incident angle *n*_0_sin*θ_sp_* of 1.50 for case (1), 1.43 for case (2), 1.44 for case (3), and 1.48 for case (4); the convergence of all the cases was reached. The numerical fluctuations between 149 diffracted orders and 151 diffracted orders were 0.28%, 0.24%, 0.17%, and 0.04%, respectively.

The convergent tests for the four extreme cases in the study showed that the diffracted order of 151 orders achieved the numerical stability of 0.28%, and the convergence was reached. Of course, the number of diffracted orders could be increased to achieve higher numerical precision; this, of course, would come with increased demand for computing time and resources. Therefore, the subsequential results were computed using 151 diffracted orders.

### 3.2. Effect of Roughness on the SPR Sensitivity

**Figure 8:**
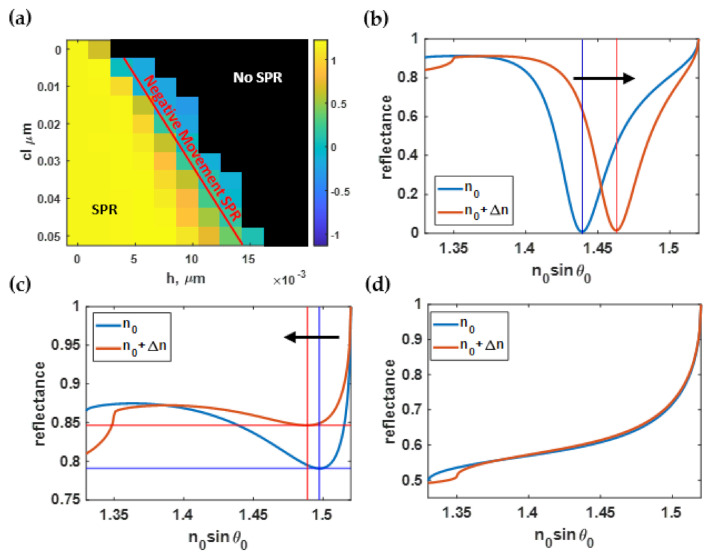
(**a**) Sensitivity calculated using Monte Carlo simulation; (**b**) SPR dips at *h* of 1 nm and *cl* of 50 nm; (**c**) negative SPR dip movement at *h* of 9 nm and *cl* of 15 nm; and (**d**) reflectance when there was no SPR dip present at *h* of 15 nm and *cl* of 5 nm.

Figure 8a shows the sensitivity calculated using Equation (3) and the RCWA simulation with the Monte Carlo model described in Section 2.2 and Section 2.3 for different roughness profiles generated as described in Section 2.1. The sensitivity depended strongly on the roughness of the gold sensor. For the smooth surface, the sensitivity was 7.47 rad·RIU^−1^/μm. The sensitivity degraded when the *cl* decreased and *h* increased. Note that the larger *cl* indicated a smoother surface. The SPR responses shown in Figure 8a were categorized into three regions labeled as ‘SPR’, ‘Negative movement SPR’, and ‘No SPR’. The SPR label was for the roughnesses for which the SPR dip responded to the change in the higher sample refractive index by the dip movement towards the larger plasmonic angle, as shown in Figure 8b. In addition, at a specific level of roughness with an *h* value ranging between 4 and 8 nm, the sensitivity slightly increased to just above 7.50 rad·RIU^−1^/μm. On the other hand, there was a region where the plasmonic angle moved slightly towards a lower coupling incident angle, such as at *h* of 9 nm and *cl* of 15 nm, as shown in Figure 8c. The third region was labeled ‘No SPR’; no plasmonic dip appeared in the reflectance spectra, as shown in Figure 8d.

**Figure 9:**
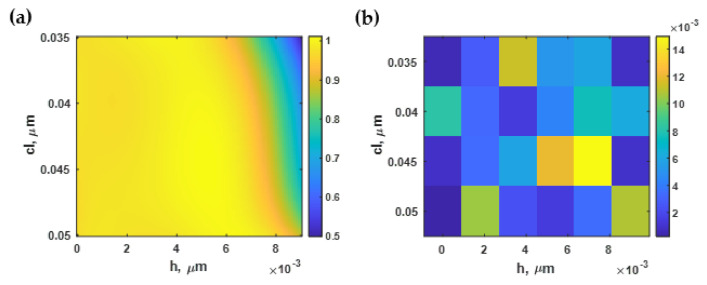
(**a**) The normalized sensitivity based on Equation (8); and (**b**) the difference between Equation (8) and the RCWA simulation.

The sensitivity contour shown in Figure 8a could then be selected based on the area of interest, with *h* and *cl* ranging from 0 nm to 9 nm and 35 nm to 50 nm, respectively. It was then normalized to the maximum sensitivity and the curve fitted to the fifth-degree polynomial function *S(h, cl)* using a built-in curve-fitting toolbox in MATLAB. The curve-fitted model expressed in Equation (8) had a coefficient of determination R^2^ of 99.80%. Figure 9a shows the curve-fitted contour calculated using Equation (8). Note that the normalized sensitivity was curve-fitted using different polynomial degrees ranging from the 2nd-order to the 5th-order polynomial function, and calculated the R^2^ for each polynomial curve-fitting function. The reason for limiting to the 5th order polynomial was that higher orders could distort the shape of the fitted curve by fitting with high-frequency components, including noise artifacts in the model. The general practice for curve fitting is to start with low polynomial order and calculate the corresponding R^2^ and absolute or root-mean-square error between the fitted contour and the curve-fitting function. The error should appear as random noise with no noticeable shapes and patterns, as shown in Figure 9b. Then, the optimal polynomial order can be tested by curve-fitting the contour with a higher-order polynomial and determining error. The higher-order polynomial can be employed before the error contour begins to form a noticeable pattern; in other words, a distortion between the contour and the curve-fitting model. Here, the fifth-degree polynomial function was chosen since it provided the highest R^2^ value. The curve-fitted equation was then validated by calculating the absolute error, comparing the normalized sensitivity contour calculated using RCWA to the parameter calculated using the curve-fitted equations as shown in Figure 9b. The maximum error shown in Figure 9b was 0.015, which was well below 1.5%. The curve-fitting procedure was also adopted to model the other performance parameters in the subsequent sections.

(8) $$\begin{array}{cc} S(h,cl)=-1.31 & +4348h+161.3cl-\left(8.11\times 10^{5}\right)h^{2}-\left(3.02\times 10^{5}\right)hcl\\ & -3751cl^{2}+\left(8.38\times 10^{6}\right)h^{3}+\left(5.54,\times ,10^{7}\right)h^{2}cl\\ & +\left(6.88\times 10^{6}\right)hcl^{2}+\left(2.88,\times ,10^{4}\right)cl^{3}+\left(5.10\times 10^{7}\right)h^{4}\\ & -\left(5.24\times 10^{8}\right)h^{3}cl-\left(1.23\times 10^{9}\right)h^{2}cl^{2}\\ & -\left(5.15\times 10^{7}\right)hcl^{3}+\left(2.04\times 10^{10}\right)h^{5}\\ & -\left(1.47\times 10^{10}\right)h^{4}cl+\left(9.77\times 10^{9}\right)h^{3}cl^{2}\\ & +\left(8.64\times 10^{9}\right)h^{2}cl^{3} \end{array}$$

The normalized sensitivity contour in Figure 9a shows that the roughness in the range *cl* of 35 nm to 50 nm and the *h* of 0 nm to 9 nm did not affect the plasmonic dip movement much. On the other hand, the roughness outside this regime dramatically degraded the sensitivity, and the roughness height *h* affected the dip movement more than the roughness period. In the next section, the FWHM will be analyzed to quantify the effect of the roughness on the FWHM. A sensitive sensor requires high sensitivity and a narrow FWHM, based on Equation (7).

### 3.3. Effect of Roughness on the SPR Full Width at Half Maximum (FWHM)

**Figure 10:**
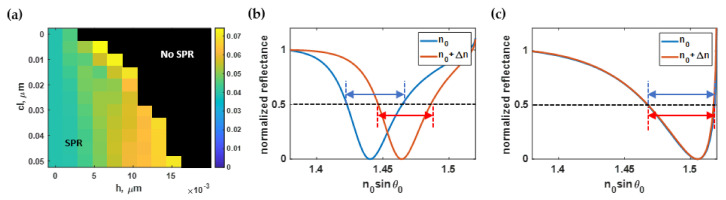
(**a**) The full width at half maximum in rad/μm calculated using Monte Carlo simulation, (**b**) SPR dips at *h* of 1 nm and *cl* of 50 nm with an average FWHM of 0.04 rad/μm; and (**c**) SPR dips at *h* of 7 nm and *cl* of 15 nm with an average FWHM of 0.05 rad/μm.

Figure 10a showed the average FWHM of the SPR reflectance dip when the refractive index was 1.33 and 1.35, as calculated using Equation (4) and the methods described in Section 2.1, Section 2.2 and Section 2.3 for different roughness profiles. The narrowest SPR dip occurred at *h* of 1 nm and *cl* of 50 nm, as shown in Figure 10b. The *h* parameter had a more substantial effect on the FWHM than the *cl* parameter, as shown in Figure 10a. Figure 10c shows widened SPR dips due to the surface roughness *h* of 7 nm and *cl* of 15 nm.

**Figure 11:**
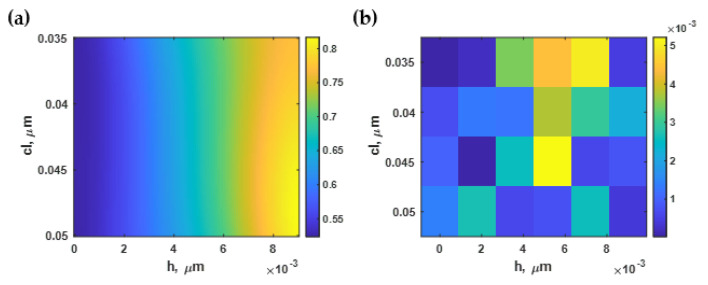
(**a**) The normalized full width at half maximum using Equation (9); and (**b**) the difference between Equation (9) and the RCWA simulation.

The region of interest of the *FWHM* contour illustrated in Figure 10a was normalized to its maximum value and curve-fitted to the polynomial function *FWHM*(*h, cl*) using the built-in curve-fitting toolbox in MATLAB. Figure 11a shows the curve-fitted contour calculated based on Equation (9). The R^2^ of the curve-fitted model was 99.95%. In addition, the absolute values of the residue between the equation-based model and the RCWA simulations are shown in Figure 11b.

(9) $$\begin{array}{cc} FWHM(h,cl)= & 0.71+403.5h-14.1cl-\left(1.75\times 10^{4}\right)h^{2}-\left(2.51\times 10^{4}\right)hcl\\ & +364.5cl^{2}-\left(4.09\times 10^{6}\right)h^{3}+\left(2.10\times 10^{6}\right)h^{2}cl\\ & +\left(4.90\times 10^{5}\right)hcl^{2}-3101cl^{3}+\left(5.93\times 10^{8}\right)h^{4}\\ & -\left(7.61\times 10^{7}\right)h^{3}cl-\left(2.90\times 10^{7}\right)h^{2}cl^{2}-\left(2.94\times 10^{6}\right)hcl^{3}\\ & -\left(1.78\times 10^{10}\right)h^{5}-\left(4.36\times 10^{9}\right)h^{4}cl+\left(1.98\times 10^{9}\right)h^{3}cl^{2}\\ & -\left(2.23\times 10^{7}\right)h^{2}cl^{3} \end{array}$$

The curve-fitted contour in Figure 11a indicated that the FWHM was not affected by the roughness period *cl*; on the other hand, the FWHM was mainly affected by the roughness height *h*. The *h* below 1 nm did not degrade the sensitivity or the FWHM. The RMS roughness extracted from reported fabrication processes was around 1 nm, as shown in Table 1. If the dentition of FOM were only the sensitivity over the FWHM, as usually employed in several articles [69,70], the FOM of the smooth gold sensor and the gold sensor with the roughness of *cl* of 50 nm and *h* of 1 nm were 30.49 RIU^−1^ and 29.98 RIU^−1^, respectively. In other words, an ideally smooth surface would perform similar to a gold sensor fabricated using the reviewed coating and surface treatment processes, with only a 1.7% difference in sensing performance. Hence, one may conclude that a surface treatment after the coating process is not necessary. Of course, the sensitivity over the FWHM cannot provide a complete story without considering the optical intensity and the signal-to-noise ratio. Since the SPR measures reflectance through attenuated total internal reflection, reflectance spectra usually have a strong background. If the plasmonic dip intensity is not deep, the optical detection measures a weak signal over a strong background, leading to a weak signal-to-noise ratio. Therefore, here we introduced the FOM as expressed in (7). The change in plasmonic dip intensity and the optical signal contrast are investigated and quantified in the following two sections. It will be shown later that the proposed FOM formula could provide a realistic refractive index sensing performance assessment.

### 3.4. Effect of Roughness on the Intensity Contrast (ΔI)

**Figure 12:**
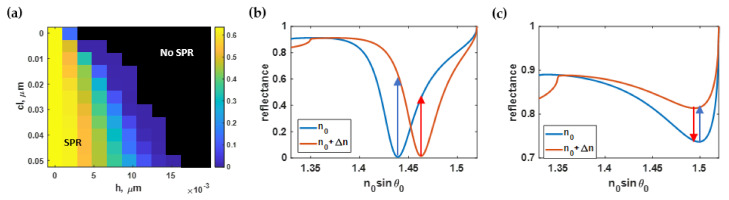
(**a**) The change in intensity calculated using Monte Carlo simulation; (**b**) SPR dips at *h* of 1 nm and *cl* of 50 nm with an average intensity difference of 0.62; and (**c**) and SPR dips at *h* of 7 nm and *cl* of 15 nm with an average intensity difference of 0.004.

Figure 12a shows the average intensity change Δ*I* of the simulated reflectance as the refractive index of the sensing area was altered from 1.33 to 1.35 calculated using Equation (5) for the different constructed rough surface profiles. The surface plasmon resonance with the highest intensity contrast occurred at *h* of 1 nm and *cl* of 50 nm, as illustrated in Figure 12b. The intensity difference parameter was significantly reduced as the *h* increased and *cl* decreased. At a rougher surface *h* of 7 nm and *cl* of 15 nm, the plasmonic intensity of *n_s_* of 1.33 was lower than the *n_s_* of the 1.35 case, as depicted in Figure 12c.

**Figure 13:**
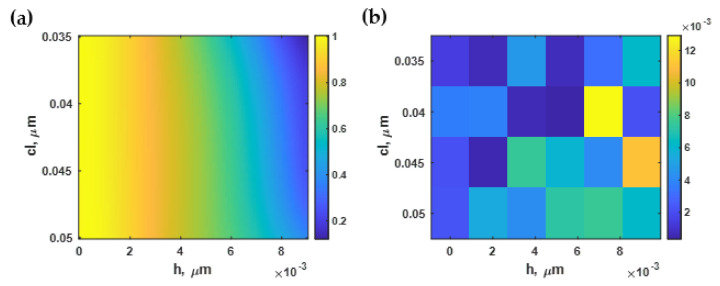
(**a**) The normalized intensity difference using Equation (10); and (**b**) the difference between Equation (10) and the RCWA simulation.

The curve-fitting toolbox in MATLAB has then been employed to curve-fit the region of interest of the normalized intensity difference in Figure 12a to the fourth-degree polynomial function Δ*I(h, cl)* with an R^2^ of 99.86%. Figure 13a,b show the contour plot based on the curve-fitted Equation (10) and the absolute values of the residue between the equation-based model and the RCWA model, respectively.

(10) $$\begin{array}{cc} \Delta I(h,cl)=2.78 & -1882h-123.3cl+8375h^{2}+\left(1.27\times 10^{5}\right)hcl+2804cl^{2}\\ & +\left(3.19\times 10^{6}\right)h^{3}-\left(1.79\times 10^{6}\right)h^{2}cl-\left(2.79\times 10^{6}\right)hcl^{2}\\ & -\left(2.09\times 10^{4}\right)cl^{3}-10^{8}h^{4}-\left(3.61\times 10^{7}\right)h^{3}cl\\ & +\left(2.94\times 10^{7}\right)h^{2}cl^{2}+\left(1.99\times 10^{7}\right)hcl^{3} \end{array}$$

Similar to the sensitivity and the FWHM, the optical intensity contrast at the plasmonic dips was mainly affected by the roughness height, not the roughness period. The lower roughness height had a stronger optical contrast, which dramatically declined when the roughness height was more than 8 nm. However, when the *h* was below 2 nm, the intensity contrast decreased by only 10%, as depicted in Figure 13a. In the next section, the optical intensity at the plasmonic angle is analyzed.

### 3.5. Effect of Roughness on the SPR Dip Intensity (I_sp_)

**Figure 14:**
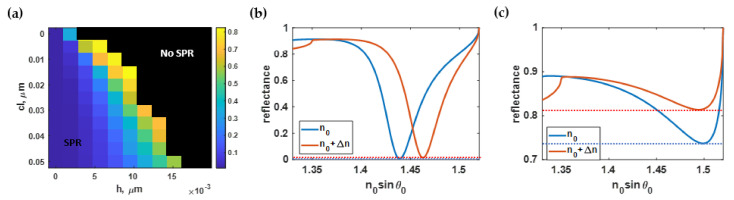
(**a**) The dip intensity calculated using Monte Carlo simulation; (**b**) SPR dips at *h* of 1 nm and *cl* of 50 nm with an average dip intensity of 0.01; and (**c**) SPR reflectance spectrum at *h* of 5 nm and *cl* of 10 nm with an average dip intensity of 0.78.

The average dip intensities *I_sp_* calculated using Equation (6) for different rough surfaces of the computed surface plasmon resonance spectrum with the refractive index of 1.33 and 1.35 are shown in Figure 14a. Similar to the full width at half maximum, the average roughness *h* had a more substantial effect on the dip intensity than *cl*, as illustrated in Figure 14a. The smoothest simulated surface (*h* and *cl* equal to 1 nm and 50 nm, respectively) gave the lowest dip intensity, as displayed in Figure 14b, and dramatically escalated as the roughness increased (Figure 14c).

**Figure 15:**
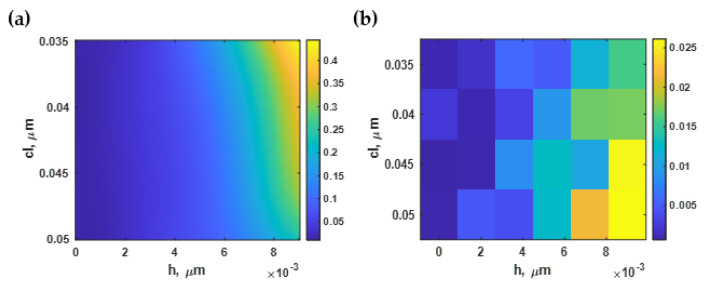
(**a**) The normalized dip intensity using Equation (11); and (**b**) the difference between Equation (11) and the simulated output.

The area of interest of the dip intensity contour displayed in Figure 14a was then normalized to the maximum value and then curve-fitted to the fifth-degree polynomial function *I_sp_(h, cl)* with an R^2^ of 99.82% using the built-in curve-fitting toolbox in MATLAB. The curved-fitted model, expressed in Equation (11), was then employed in the contour plot shown in Figure 15a. Finally, the absolute residue between the RCWA simulated contour and the equation-based contour is illustrated in Figure 15b.

(11) $$\begin{array}{cc} I_{sp}(h,cl)= & -1.77+1304h+125.4cl-\left(1.21\times 10^{4}\right)h^{2}-\left(8.94\times 10^{4}\right)hcl\\ & \;-2899cl^{2}+\left(2.86\times 10^{5}\right)h^{3}+\left(8.93\times 10^{5}\right)h^{2}cl\\ & \;+\left(2.01\times 10^{6}\right)hcl^{2}+\left(2.21\times 10^{4}\right)cl^{3}-\left(2.88\times 10^{7}\right)h^{4}\\ & \;+\left(1.13\times 10^{7}\right)h^{3}cl-\left(1.48\times 10^{7}\right)h^{2}cl^{2}-\left(1.47\times 10^{7}\right)hcl^{3} \end{array}$$

The optical intensity at the plasmonic angle was also affected mainly by the *h*, not the *cl* like the sensitivity, the FHWM, and the intensity contrast quantified in the earlier sections. The smoother surface provided a deeper intensity dip. However, the change in the optical intensity dip was within only 3%. It will be shown later in the next section that although these performance parameters slightly changed within a roughness range of 2 nm, these performance degradations could accumulate, resulting in the reduced FOM performance in Equation (7) by 50%, reflecting the experimental sensing performance of surface plasmon sensors with different roughnesses reported in Agarwal et al. [51].

### 3.6. Effect of Roughness on the SPR Figure of Merit (FOM)

**Figure 16:**
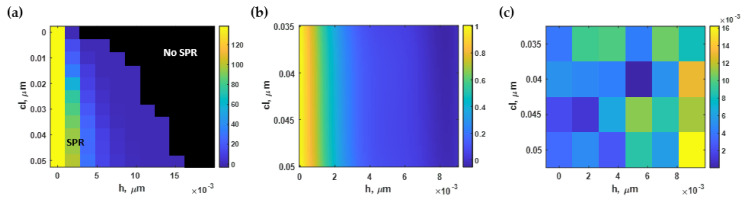
(**a**) The FOM using Monte Carlo simulation; (**b**) the normalized FOM based on Equation (12); and (**c**) the difference between Equation (12) and the RCWA simulation.

The illustration of the FOM of the surface plasmon resonance spectrum calculated using Equation (7) and the procedure described in Section 2.1, Section 2.2 and Section 2.3 for different roughness is shown in Figure 16a. The FOM decreased as the *h* increased and the *cl* decreased. In other words, the rougher surface led to the worse refractive index sensing capability. This contour was then normalized to the maximum calculated FOM and curve-fitted to polynomial function *FOM(h, cl)*, as expressed in Equation (12), with an R^2^ of 99.98%, using the built-in curve-fitting toolbox in MATLAB. Figure 16b shows the curve-fitted contour based on the curve-fitting model. According to Figure 16c, the model had significantly high accuracy, with the maximum absolute residue between Equation (12) and the RCWA simulated contour below 1.6%.

(12) $$\begin{array}{cc} FOM(h,cl)=-2 & -1181h+214cl+(1.70\times 10^{5})h^{2}+\left(5\times 10^{4}\right)hcl\\ & -5038cl^{2}+\left(2.06\times 10^{7}\right)h^{3}-\left(1.14\times 10^{7}\right)h^{2}cl\\ & -\left(9.68\times 10^{5}\right)hcl^{2}+\left(3.92\times 10^{4}\right)cl^{3}-\left(4.61\times 10^{9}\right)h^{4}\\ & +\left(4.89\times 10^{8}\right)h^{3}cl+\left(1.89\times 10^{8}\right)h^{2}cl^{2}+\left(6.93\times 10^{6}\right)hcl^{3}\\ & +\left(2.48\times 10^{11}\right)h^{5}-\left(2.11\times 10^{10}\right)h^{4}cl-\left(9.18\times 10^{8}\right)h^{3}cl^{2}\\ & -\left(1.34\times 10^{9}\right)h^{2}cl^{3} \end{array}$$

The roughness height strongly affected the FOM shown in Figure 16a; meanwhile, the roughness period did not degrade the FOM performance. For example, for a roughness height of 2 nm, the FOM parameter decreased by 50%. Next, we adopted the analysis to quantify the literature’s expected refractive index sensing performance of different deposition and surface smoothing techniques.

### 3.7. Plasmonic Sensing Responses of Different Depositions and Surface-Treatment Technologies

Table 2 shows the performance parameters calculated for the idealized smooth surface and different plasmonic gold sensor roughness reported in the literature for different deposition technologies and surface treatments calculated using Equations (8)–(12). The surface RMS reported in the literature were extracted from each of the referenced articles in Table 2 and then converted to corresponding *h* and *cl* values using Equation (2), assuming the *h* and *1/cl* had a similar length, as reported by Agarwal et al. [51]. Note that from the above analysis, the *cl* did not affect the sensitivity, the FWHM, the intensity contrast, or the FOM much compared to the roughness height, as discussed in the earlier result sections. For the sensitivity, all the gold surfaces had a similar performance, indicating the roughness did not affect how far the plasmonic dip moved. For the plasmonic angle, the plasmonic dip for the ideally smooth surface had the lowest plasmonic angle of 71.40°, the same as the other treatment methods with an RMS of less than 1 nm. The sputter coating with no additional treatment method, chemically grown single-crystalline gold, and thermal annealing had a slightly larger plasmonic angle. The FWHM was narrowest for the smooth surface and slightly increased for other coating and treatment techniques. The Δ*I* performance was similar for all the cases, implying that there was no need for additional surface treatment for the SPR measurement relying on measuring the change in intensity. The intensity dip at the plasmonic angle was strongly affected by the surface roughness, increasing approximately 3.5 times for the nontreated surface compared to the ideally smooth surface. For the FOM calculated using Equation (7), the FOM for the smooth surface was 139.16 and degraded to 90.09 for the sputter-coating technique accounting for 35.26% FOM degradation. Thus, the surface-treatment methods could improve the FOM response for refractive index sensing applications.

## 4. Conclusions

There are challenges to overcome for SPR measurements demanding high precision and responsivity, including the environmental fluctuations and the quality of the SPR sensor. In this paper, we proposed a theoretical analysis quantifying the effect of plasmonic gold sensor roughness on its refractive index sensing capability using rigorous coupled-wave analysis. SPR sensor surface profiles with different roughness heights and roughness periods were modeled using a digital random number generator and a low-pass filter to limit the spatial frequency of the roughness to mimic the surface morphology reported in the literature. The Monte Carlo simulation was then applied to calculate the average plasmonic reflectance spectra for the roughness profiles; the sensing performance parameters, including the sensitivity, the full width at half maximum, the plasmonic angle, the plasmonic intensity at the SPR dip position, the change in optical reflectance and the figure of merit, were computed for different surface roughness profiles. Here, we also proposed and discussed a figure-of-merit definition considering the signal contrast; the proposed FOM could provide a reasonable estimation of refractive index sensing performance. The analysis agreed with the experimental results for different roughness levels reported in the literature. The performance of surface-plasmon-based sensors can be significantly affected by the roughness height. Different coating technologies and surface-smoothing techniques were analyzed and discussed. The RMS roughness reported in the literature for different deposition and surface treatment technologies were employed and analyzed using the proposed theoretical framework.

In the comparison of the conventional sputter-coated SPR-based sensor with a root-mean-square roughness of 1.2 nm to an ideal smooth surface sensor, the sensitivity, plasmonic angle, full width at half maximum, and intensity at the plasmonic dip increased by 1.18%, 0.16%, 3.61%, and 3.51 times, respectively; while the intensity difference at the plasmonic angle and the figure of merit degraded by 6.04% and 35.26%, respectively. Therefore, for applications demanding high precision and responsivity, SPR sensors prepared by conventional deposition methods alone, such as sputter coating, are insufficient to achieve high refractive index sensing performance. Therefore, it is recommended to post-process the SPR sensors using one of the analyzed surface smoothing techniques.

## Figures and Tables

**Table 1:** Shows surface roughness and surface smoothing methods.

Method	Remaining Root Mean Square (RMS) Roughness
No treatment: sputter coating	RMS = 1.2 nm
Chemical polishing [25,26]	RMS = 0.38 ± 0.05 nm
Mica substrate utilizing [27]	RMS = 0.2 nm
Chemically grown single-crystalline gold [31]	RMS < 1 nm
Laser ablation [32]	RMS = 0.17 nm
Helium ion beam [33]	RMS = 0.267 nm
Thermal annealing [34]	RMS < 1 nm

**Table 2:** Refractive index sensing parameters for different surface roughness and smoothing methods.

Method	RMS Roughness (nm)	S (rad · RIU^−1^/μm)	*θ_sp_* (Degree)	FWHM (rad/μm)	ΔI	I_sp_	FOM
Ideal smooth surface	0	7.46	71.40	0.039	0.64	0.007	139.16
No treatment: sputter coating	1.2	7.55	71.51	0.040	0.60	0.024	90.09
Chemical polishing [25,26]	0.38	7.52	71.40	0.039	0.63	0.013	125.20
Mica substrate utilizing [27]	0.2	7.50	71.40	0.039	0.64	0.010	132.16
Chemically grown single-crystalline gold [31]	<1.0	>7.55	<71.51	<0.040	>0.61	<0.021	>98.77
Laser ablation [32]	0.17	7.50	71.40	0.039	0.64	0.010	133.27
Helium ion beam [33]	0.267	7.51	71.40	0.039	0.63	0.011	129.63
Thermal annealing [34]	<1.0	>7.55	<71.51	<0.040	>0.61	<0.021	>98.77

## Data Availability

Not applicable.
